# Translation, transcultural adaptation, and validation of two questionnaires on shared decision making

**DOI:** 10.1111/hex.12842

**Published:** 2018-10-17

**Authors:** María Victoria Ruiz Yanzi, Mariela Silvia Barani, Juan Victor Ariel Franco, Fernando Ramón Vazquez Peña, Sergio Adrian Terrasa, Karin Silvana Kopitowski

**Affiliations:** ^1^ Family and Community Medicine Service Hospital Italiano de Buenos aires Buenos Aires Argentina

**Keywords:** Argentina, patient participation, psychometrics, shared decision making, surveys and questionnaires

## Abstract

**Objective:**

To translate, transcultural adapt, and validate the “CollaboRATE” measure and the “Ask 3 Questions” intervention in Argentina, allowing us to quantify the degree of use and implementation of shared decision making (SDM).

**Design:**

Cross‐sectional study.

**Setting and Participants:**

Data were collected in an academic hospital in Buenos Aires. Physician–patient pairs were invited to participate following their scheduled outpatient visits.

**Measurements:**

Two processes were carried out as follows: (a) The translation and transcultural adaptation process, in which translations were produced and then adapted to Spanish. (b) The validation process, in which questionnaires were completed by patient–physician pairs, the results of which were subsequently analysed. Reliability (Cronbach's alpha) and construct validity (principal component analysis) were assessed.

**Results:**

The final Spanish versions of “CollaboRATE” and “Ask 3 Questions” were tested in a primary care sample of 56 participants. Both instruments presented adequate reliability and validity. Internal consistency yielded a Cronbach's alpha of 98.3 for the “CollaboRATE” measure and 0.77 and 0.69 for the patients and physicians versions of the “Ask 3 Questions” intervention. Principal components analysis showed eigenvalues of first component >1.

**Discussion and Conclusions:**

We obtained valid and reliable Spanish versions of the “CollaboRATE” measure and the “Ask 3 Questions” intervention. These versions can be used for the assessment of SDM in clinical visits, and to obtain new information which could help the monitoring of its implementation.

## 1 INTRODUCTION

*Shared Decision Making* (SDM) is a process in which health‐care teams work together with patients to make preventive, diagnostic and therapeutic health decisions based on the best clinical evidence available, while at the same time maintaining the patients well‐informed and respecting their preferences and values.^1^, ^2^, ^3^, ^4^, ^5^

Evidence suggests that patients who become involved in the decision‐making process regarding their health‐care obtain better results and experience more satisfaction with the overall care experience than those who take a more passive role.^6^, ^7^, ^8^ A Cochrane review of 115 studies (including a total of over 34 000 patients) concluded that there is sufficient evidence to suggest that SDM contributes to a better understanding on the part of patients with respect to the different options offered by their health‐care provider.^9^

The growing interest in SDM has been accompanied by an increased need to assess this process. The current state of instruments capable of measuring SDM represents a significant challenge to continued research on SDM and implementation of SDM strategies. For instance, Scholl et al^10^ alone have identified 29 different scales attempting to measure this construct.

A close look at the literature reveals that these instruments can be divided into two principal subcategories: (a) those that seek the most “objective” assessment possible, through the participation of external observers;^11^, ^12^, ^13^, ^14^, ^15^ and (b) those that emphasize the perceptions of health‐care users^16^, ^17^, ^18^ (some of these also include the perceptions of health‐care providers).

The overwhelming majority of *instruments that emphasize the perception of health‐care users* are self‐administered (by the patients themselves) and quite extensive, which makes their implementation difficult in clinical settings. In this sense, the length of these instruments difficults their routinely use in clinical practice limiting timely feedback to health‐care providers, which would help them modify their practice.^10^

In order to address this issue, Elwyn et al^19^ developed the CollaboRATE measure with the aim to develop a short, practical and reliable instrument that could provide a more immediate feedback to health‐care providers. CollaboRATE demonstrated concurrent validity with other SDM measures, excellent intrarater reliability and sensitivity to change.^20^ This instrument was meant to be general enough to be immediately applied following diverse types of outpatient clinic visits.

It should be noted that the outcomes of different studies on SDM have generated inconsistent results, perhaps due to the fact that these two subcategories of instruments (external observers vs user perceptions) do not assess the exact same constructs.^17^

In this context, Shepherd et al^21^ developed an intervention to measure the level of SDM involvement in each clinical visit and was used as part of the MAGIC programme (Making Good Decisions in Collaboration) in the UK, taking into account both perspectives (that of the patient and of the health‐care provider). This intervention, called “Ask 3 Questions,” consists of brief, self‐administered questionnaires to be completed immediately following the clinical visit in question, designed to reliably measure the same construct for both parties involved—one for the patient, one for the physician.

Asking these three questions improved information given by family physicians and increased physician facilitation of patient involvement using the Assessing Communication about Evidence and Patient Preferences (ACEPP) tool and the OPTION tool.^21^

There are some studies that suggest that SDM (measured with other tools) might be associated with better outcomes related to decisional conflict and patient satisfaction but it is unclear whether this relates to clinical outcomes.^22^, ^23^, ^24^, ^25^

We believe that the CollaboRATE and the “Ask 3 Questions” have key features that we consider important for a shared decision‐making instrument: they are short and take little time to apply, allowing a timely feedback. The questions are open and subjective enough to be used in diverse settings, and they were developed by research groups that have experience in the development of other, lengthier, instruments.^12^ Therefore, we think this might be the kind of tools needed to assess SDM.

## 2 RESEARCH RATIONALE

Although a number of articles have been published reflecting on the notion of SDM,^26^, ^27^ we were unable to find any that assessed the implementation of SDM in Argentina or that examined physicians’ or patients’ levels of SDM involvement.^28^

Therefore, we concluded that the translation, transcultural adaptation and validation of the “CollaboRATE” and “Ask 3 Questions” measures—given their manageable length, fast application and possibility for immediate feedback—would allow us to quantify the degree of use and implementation of SDM in our practice. These measurements could be useful in the monitoring of policies to increase SDM uptake and health‐care quality in Argentina and Latin America.

## 3 OBJECTIVES

To translate into Spanish, transcultural adapt, and validate the CollaboRATE measure and the “*Ask 3 Questions*” intervention in Argentina.

## 4 METHODS

### 4.1 Instruments

Three instruments were used as follows: the two original “Ask 3 Questions” *intervention* (one for physicians and the other for patients), used *by the MAGIC programme* of the National Institute of Health, UK, and developed by Shepherd et al^20^ at the University of Sydney, and the CollaboRATE patient measure, developed by Glyn Elwyn et al^19^ of the Dartmouth Center for Health Care Delivery Science in the United States.

### 4.2 Overall design

Two processes were carried out as follows:

- The translation and transcultural adaptation process, in which translations were produced and then adapted to the variant of Spanish used in Argentina. The research team participated in this process and carried out cognitive interviews.
- The validation process, in which 56 questionnaires were distributed to patient–physician pairs and completed, the results of which were subsequently analysed.

Both processes are described below.

### 4.3 Phase 1: translation process

The *first step* was to solicit the services of two independent translators (of whom one worked in the medical field while the other did not) in order to do a preliminary English‐to‐Spanish translation. Spanish was the native language of both translators. Two Spanish versions of the measure and intervention were obtained, which a committee then compared, evaluated and synthesized into one preliminary Spanish version that included what experts thought would represent better the meaning of the original questions and the understanding of patients, taking into account the opinions of the non‐medical translator. (“*preliminar_1*”). This committee was composed by the two translators, the lead researcher and three SDM experts.

As part of the *second step*, a back‐translation of the “*preliminar_1*” version was done. The same methodology was employed, the only difference being that in this step the translators were both native English speakers. Two back‐translated English versions were obtained.

The *third step* consisted of assessing, comparing and synthesizing a final version (“*preliminar_2*”) from all the preliminary versions (the original questionnaire, the “*preliminar_1*” version, and both English back‐translations). This was carried out by a committee composed of the lead researcher and three SDM experts. From this process, the “*preliminar_3*” version was obtained.

### 4.4 Phase 2: transcultural adaptation process

The *fourth step* involved administering the “*preliminar_3*” version to nine patients selected by a convenience sample, with the objective of assessing its viability and applicability. Respondents were recruited in the waiting rooms of the outpatient clinics at the Hospital Italiano de Buenos Aires and a public Primary Care Facility that provides health‐care services to low‐income populations (Centro de Salud San Pantaleón in the Bajo Boulogne neighbourhood of San Isidro, at the outskirts of Buenos Aires).

The interviews were divided into three parts: (a) initially, demographic data were collected and respondents were informed of the study's confidentiality; (b) the respondent then proceeded to complete the questionnaire; and finally (c) the cognitive interview, in which the researcher reviewed the questionnaire with the respondent, inquiring about any points that might have generated difficulties or were unclear while completing the questionnaire and at the same time, assessing the respondent's comprehension of the questions.

The instruments were reread after completion by the investigator, leaving time for the expressions of concerns, doubt or thoughts by the interviewee.

The *fifth and final step* was to use the information obtained in the interviews to formulate the “FINAL” versions of the three questionnaires, which had been translated and transcultural adapted to the variant of Spanish spoken in Argentina.

The research team decided to undertake a process of transcultural adaptation of the instruments in addition to their translation into Spanish. This decision was based on the conclusion that in order to best adapt them to the variant of Spanish spoken in Argentina, the careful choice of words corresponding to the country's lexical particularities (especially in the case of Buenos Aires) would be necessary in order to obtain better results than would a version with idiomatic expressions and “standard” varieties of language use prevalent in other Spanish‐speaking regions.

### 4.5 Cognitive interviews: recruitment process

Patients were recruited in the waiting rooms of the outpatient clinics at the Hospital Italiano de Buenos Aires and in the waiting rooms of the Centro de Salud San Pantaleón. Cognitive interviews were conducted until saturation was reached.

The Hospital Italiano de Buenos Aires is an academic hospital located in Buenos Aires, which offers a private insurance plan and predominantly serves a middle‐income population with higher levels of education, who reside in urban and suburban areas.

The Centro de Salud San Pantaleón is in the Bajo Boulogne neighbourhood of the Municipality of San Isidro, at the outskirts of Buenos Aires. This health centre offers free health‐care services to a primarily low‐income population with lower educational levels, who tend to reside in the nearby community.

The interviews were conducted in these two locations to assure that the questionnaires could be successfully interpreted by respondents with varying levels of education.

### 4.6 Validation process

We used the same methodology to validate the three questionnaires. Between September and December of 2015, 56 clinical outpatient visits were selected, 30 of which comprised the area of family medicine and primary care (with primary care physicians); the remaining 26 were visits with specialists (endocrinology, dermatology, general surgery, pulmonology and arterial hypertension).

#### 4.6.1 Population

Physician–patient pairs were invited to participate following their scheduled visits at the outpatient clinics for clients of the private insurance plan of the Hospital Italiano de Buenos Aires.

#### 4.6.2 Sample

We based our decision regarding the final sample size of 56 on the consensus found in the specialized literature which indicates that if n is <100, there should be at least 10 completed surveys per item,^29^ and n should never be smaller than 50.^30^

#### 4.6.3 Clinical visit selection process

Two times a week over the course of 2 months, the lead researcher visited the outpatient clinic to administer the “CollaboRATE” measure and the “Ask 3 questions” intervention. Depending on the time of day, she recruited respondents either on the first or the second floor of the clinic (primary care physicians attend on the first floor, whereas specialists have their offices in the second floor).

Between three and eight questionnaires were administered each day to physician–patient pairs according to the following procedure:

The lead researcher invited the patient to anonymously participate in the study after her/his health visit was completed. Once a patient agreed to participate in the study and completed the intervention, the researcher proceeded to invite the patient's physician to complete them as well. The patient and the physician completed their respective measure and intervention in separate rooms, as soon as the visit ended, so they could both remember it (physicians are usually inside their offices and patients in the waiting room). Neither party was informed of the other's responses. Instruments were given to patient–physician pairs only. Physicians knew that patients were asked to participate, but they did not know which patients participated in the research, although only three patients refused to participate.

Once every part was completed (two by the patient and one by the physician), the lead researcher collected them and proceeded to wait for the next clinical visit to conclude, repeating the process and inviting another patient–physician pair to participate. No patient participated more than once. The study's final results only included the instruments that had been completed both by the physician and their patient. No physician refused to participate, and three patients refused to participate. Three physicians participated twice, and no participants participated in more than one pair.

#### 4.6.4 Construct validity

Construct validity was evaluated by a principal component analysis (PCA), taking into account the total variance and the adequacy of a unidimensional model.^25^, ^31^ This was based on the assumption that each item on the questionnaires load on a single factor. Sampling adequacy was determined using the following measures (see Table 1): (a) Bartlett's sphericity test, which at significant levels expresses strong correlation among the included variables,^25^ indicating that factor analysis is appropriate; (b) Keyser, Meyer and Olkin (KMO) values, which were found to be acceptable (>0.7)^26^ for the “CollaboRATE” measure and “Ask 3 Questions” intervention(for patients). Regarding the “Ask 3 Questions” intervention for physicians, given that the KMO value was acceptable albeit slightly lower (0.62), sampling adequacy was also assessed using anti‐image correlation matrix diagonals, which set a minimum threshold of 0.5 to determine the appropriateness of exploratory factor analysis^32^ (see Table 1).

**Table 1:** Indexes of adequacy of the matrix for factor analysis

Questionnaires	“Ask 3 Questions” (patients)	“Ask 3 Questions” (physicians)	“CollaboRATE”
KMO (>0.5)	0.72	0.62	0.74
Bartlett's test (<0.001)	<0.0001	<0.0001	<0.0001
Anti‐image correlation matrix diagonal values (all values >0.5)		Yes	

KMO, Kaiser Meyer Olkin.

#### 4.6.5 Reliability

Internal consistency reliability was estimated by calculating Cronbach's alpha for each scale, considering that the minimum acceptable coefficient varies between 0.6 and 0.7,^25^ and that a value of 0.6 may be considered acceptable if there are fewer than 10 items in the scale.^33^

With respect to criterion validity, the research team hypothesized that there would be correlation (applying Pearson's coefficient) between the results obtained by the “CollaboRATE” measure (the version for patients that had already been validated) and those obtained by the “Ask 3 Questions” intervention for patients, given that both instruments were intended to measure the same construct. It was not expected that such correlation would be found with the “Ask 3 Questions” intervention for physicians.

Given that a strong inter‐item correlation was found for the “CollaboRATE” measure, we attempted to identify the most effective question from a mathematical point of view (ie, the question that generated the most complete information), by evaluating the correlation of each item vs the adjusted total excluding that item (“adjusted item total”) and Cronbach's alpha coefficient after excluding that item. Once the most effective question was identified using this method, the Pearson correlation coefficient of the item with the full‐form measure was verified.

## 5 RESULTS

### 5.1 Translation and transcultural adaptation process

Throughout the translation process, discrepancies among the translators were discussed in research committee meetings, to assure that the adaptation of each term would permit comprehension by the local population.

Regarding the “CollaboRATE” measure, the translators initially respected the syntactical structure of the original English version whenever its equivalent was acceptable in Spanish. For example, “how much effort was made” was translated as “*cuánto esfuerzo se hizo*.” However, during the cognitive interviews, the respondents noted that this phrasing was “*difficult to understand*” or “*unclear*,” and concluded that “*it would be better to specify that the question is asking how much effort was made by the physician*.” For this reason, in the final version, the research team decided to adopt a phrasing that would make clear the fact that the effort was to be attributed to the health‐care professional. Therefore, these questions were reformulated as follows: “*cuánto esfuerzo hizo el médico*” (“how much effort did the physician make”).

As for the “Ask 3 Questions” intervention, both the version for patients and the version for physicians included an explanatory statement with the first question (“did you discuss whether to give treatment or talk about which treatment to choose?”). The research team considered that this statement could bias the responses of physicians and patients to include only decisions regarding treatment. Therefore, in the Spanish version, this phrase was modified to include alternatives related to the discussion of screening and diagnostic options.

Similarly, in both versions of the intervention, the final item proved to be somewhat confusing for some of the respondents in the cognitive interviews. This item asked respondents what they considered important, which generated the question among respondents of “*important regarding what*.” The researchers considered that the ambiguity of this question was intentional in the original English version, and its intention was to leave the interviewee to “decide” what was important for them and assess whether the physician had addressed that issue in the interview, and therefore, it was not modified in the final Spanish version. The two instruments are available in the Appendix S1.

### 5.2 Validation

As discussed above, the sample was determined to be adequate for the validation of the three questionnaires (see Methods section and KMO values, Bartlett's test, and anti‐image correlation matrix diagonal values in Table 1).

#### 5.2.1 Regarding the three instruments

##### Construct validity

We verified the unidimensionality of the measure and intervention using a principal component analysis (PCA), considering the following criteria recommended in the literature (see Table 2):

**Table 2:** Validity and reliability of both instruments

Methods of analysis	Ask 3 Questions (patients)	Ask 3 Questions (physicians)	CollaboRATE
Principal component analysis (PCA)			
Eigenvalues			
1st Component	2.4	2.1	2.9
2nd Component	0.77	0.83	0.07
% of variance explained by the 1st component (>50%)	Yes 60.09%	Yes 53.06%	Yes 97.33%
Ratio of the difference between the 1st and 2nd eigenvalue and the 3rd and 4th eigenvalue	5.6	9	49
Factor loadings >0.55	100%	100%	100%
Analysis of internal consistency (reliability)			
Cronbach's alpha	0.77	0.69	98.3

1. *Kaiser criterion*: Only factors with an eigenvalue >1 in the first analysis are retained.^34^ Given that one of the limits of this method is its arbitrary nature (since a factor with an eigenvalue of 1.01 can be separated from another factor with an eigenvalue of 0.99),^26^ we show the values of the first and second eigenvalues in Table 2 to demonstrate their difference and to confirm that no second eigenvalue is close to the threshold of unity.
2. *Carmine's criterion* 35: Considers that the variance explained by the first component or factor is >40%.
3. *Hattie criterion* 36: This measure suggests that unidimensionality can be assessed by obtaining a relatively high value upon calculating the ratio of the difference between the first and second eigenvalues divided by the difference between the second and third eigenvalues. We accepted a value >3.^37^
4. *Gorsuch criterion* 38: This considers only factors with eigenvalues >1.41. Following this standard, it is likely that only components which are essential to the construct will be retained.^39^

Factor loadings were >0.55 in all cases (see Table 2). This is important to consider when reducing data,^40^ as it demonstrates how appropriate the reduction is, in that all variables attain an acceptable level of saturation with respect to the factor or factors to be retained.

##### Reliability

Promising results were obtained from the instruments with respect to their reliability. We found the following results for each instrument: 0.77 for the “Ask 3 questions” for patients, 0.69 for the “Ask 3 questions” for physicians and 98.3 for the CollaboRATE. These results were acceptable according to our predefined criteria for the Ask 3 questions intervention and excellent for the CollaboRATE. (see Table 2 and Methods section).

Below, we will discuss the individual characteristics of each questionnaire:

#### 5.2.2 Regarding the “Ask 3 Questions” intervention

##### Concurrent criterion validity

To evaluate the concurrent criterion validity of the “Ask 3 Questions” intervention for patients and for physicians, each with a length of four questions, we calculated the Pearson correlation coefficient for the mean results of both questionnaires with the mean results of the already validated “CollaboRATE” measure.^20^ We obtained a correlation of 0.365 with the version for patients, which we took to be reasonably strong for independent scales in our experience, and significant (*P* < 0.006), but not significant (*P* = 0.134) for the version for physicians (see Discussion section).

#### 5.2.3 Regarding the “CollaboRATE” measure and the use of a single question

Given the high correlation among the items on the “CollaboRATE” measure, considering the “adjusted item total” and “Cronbach's alpha if the item is eliminated,” we could verify that the element that best summarized the complete information from the questionnaire from a mathematical point of view was the second item (see Appendix S1). We verified that the Pearson correlation coefficient for this item with the mean of the items of the full‐form measure was very strong (0.994) and highly significant (<0.0001; see Discussion section).^41^

## 6 DISCUSSION

We obtained validated versions in Spanish of the CollaboRATE measure and “Ask 3 Questions” interventions that were tested in a primary care sample of 56 participants. Both instruments presented adequate reliability and construct and criterion validity.

We believe that the process of translation and transcultural adaptation has allowed us to identify what types of questions were easier for our patients to understand. In our experience, people needed more detailed information to answer the questions. As we mentioned above, we had to add a few more explanations to the “Ask 3 questions” introductory sentence to make it more understandable. Also in the CollaboRATE questionnaire, people thought questions were too open, which differed from the results of the cognitive interviews in the English version, in which this was not an issue.^19^ We believe that the open questions are important in this instrument because they give the patient the opportunity to answer according to their values and preferences, but it may pose a barrier for the Argentinian users when the openness of the questions does not allow them to give a response in a context in which patients are not usually asked to reflect on this topic. Our Spanish version has some differences when compared to the original version, especially adapted to our local context in which the physicians are most of the primary health‐care providers, and treatments are not usually discussed with patients. Additionally, the validation process allowed us to satisfactorily quantify the characteristics for which the questionnaires were originally created, such that the translated and adapted versions would accomplish the same objectives.

Regarding the results of both the measure and intervention, given that the “Ask 3 Questions” intervention had not been validated in its original language, we calculated Pearson correlation coefficients for the means of both questionnaires with the mean results of the previously validated “CollaboRATE” measure.

Conversely, the Pearson correlation coefficient between the results of the “CollaboRATE” measure and those of the physician version of the “Ask 3 Questions” intervention was found to be quite weak, which is consistent with the findings frequently discussed in the literature suggesting that the opinions of patients and physicians regarding participation in clinical visits are often dissimilar.

One of the strengths of this study was the instruments obtained as an outcome. They represent the first brief instruments to evaluate physician's assessments of the level of patient involvement in clinical visits, not only in Argentina but in the region as well.

Nonetheless, it must be pointed out that one of the weaknesses of the validation process was that it was carried out with an overwhelmingly middle‐class urban/suburban population. Therefore, the validity of the measure and intervention for other populations must be eventually assessed, such as the case of low‐income groups. Furthermore, an external reviewer highlighted that the translation is neutral enough to be used or tested in other Spanish‐speaking countries. The response rate was high. We had concerns regarding the sample size, but we followed the recommendations by the specialized bibliography. Regarding the exploratory factorial analysis of the surveys used to demonstrate unidimensionality, Streiner^29^ states: “There should be an absolute minimum of five subjects per variable, with the proviso that there are at least 100 subjects. If there are fewer than 100, then the ratio should be closer to 10:1.” Our ratio was 14:1 in the “Ask 3 questions” and 18:1 for the CollaboRATE Questionnaire.

On the other hand, Hair states^30^: “Regarding the sample size question, the researcher generally would not factor analyze a sample of fewer than 50 observations.” This was also taken into account. In regard to the reliability analysis with Cronbach's alpha, we propose the same sample requirements.^42^, ^43^ We reached an adequate sample size according to our bibliography.^29^, ^30^

Lastly, it should be noted that considering the results obtained by our study (see Results section) and taking into account questions raised in the literature,^36^ future research should evaluate whether to use the full‐form “CollaboRATE” measure, translated and validated in Spanish or to use the second question alone. This is because, from a mathematical point of view, the second question effectively summarizes the information that the full‐form instrument seeks to obtain. Even though this is suggested by statistical methods and it might relate to the fact that question 2 includes “options” (part of question 1) and “for me” (part of question 3), the full questionnaire might be more representative of the complete experience of shared decision making, especially for question 3, which highlights the need to focus on the things that matter to patients.

We would expect that our contribution in the development of these locally validated tools can allow the Spanish‐speaking researchers and other stakeholders when assessing the implementation of SDM. We understand that it might be necessary to conduct further research to assess the transferability of these tools in other regions in Latin America and for individuals living in low‐income settings.

## 7 CONCLUSIONS

The purpose of this study was to obtain the translated and culturally adapted versions of the “CollaboRATE” measure and “Ask 3 Questions” intervention and validate them in Argentina. The instruments appear to have been accepted by local respondents, confirming their flexibility in adapting to local realities.

This allows us to conclude that we have obtained valid and reliable Spanish versions of these instruments. At the same time, these versions can be used for the assessment of Shared Decision Making in clinical visits, and to obtain new information which could help implementation of Shared Decision Making.

## CONFLICT OF INTEREST

The authors do not present any conflict of interest.

## ETHICAL CONSIDERATIONS

The research protocol was approved by the Institutional Review Board of the Hospital Italiano de Buenos Aires, which determined that it was not necessary to obtain written consent to conduct the research, but oral consent was sufficient (File Number 2363). To obtain oral consent, the patients invited to participate in the study were given an informative text explaining the study's aims and guaranteeing that the data obtained from interviews would be kept strictly confidential.
