# Supplementary material for: Translation, transcultural adaptation, and validation of two questionnaires on shared decision making
Source: Health Expect. 2018 Oct 17;22(2):193–200. doi: 10.1111/hex.12842 (PMC6433308; doi:10.1111/hex.12842)
Supplement: Supplementary file 1 [file HEX-22-193-s001.docx]

**Respecto a la consulta que acaba de tener**

**COLLABORATE**

- **¿Cuánto esfuerzo hizo el médico para ayudarle a entender los temas relacionados con su salud?**

**0 1 2 3 4 5 6 7 8 9**

Ningún esfuerzo Todo el esfuerzo posible

- **¿Cuánto esfuerzo hizo el médico para escuchar lo que a usted más le importa sobre sus temas de salud?**

**0 1 2 3 4 5 6 7 8 9**

Ningún esfuerzo Todo el esfuerzo posible

- **Cuánto esfuerzo hizo el médico para incluir lo que a usted más le importa al momento de tomar decisiones en relación a su salud?**

**0 1 2 3 4 5 6 7 8 9**

Ningún esfuerzo Todo el esfuerzo posible

**MAGIC**

- **¿Se tomó alguna decisión en relación al cuidado de la salud del paciente o su tratamiento?** Por ejemplo, se discutió sobre dar o no tratamiento, qué tratamiento elegir, qué estudio diagnóstico o de rastreo realizar.

SI - NO

- **¿Le informó al paciente las diferentes opciones disponibles?**

SI - NO SÉ - NO

- **¿Le informó al paciente acerca de los beneficios y riesgos de cada opción?**

SI - NO SÉ - NO

- **¿Le informó al paciente cuán probables son esos beneficios y riesgos?**

SI - NO SÉ - NO

- **¿Le preguntó al paciente qué considera importante?**

SI - NO SÉ - NO

**MAGIC**

- **¿Se tomó alguna decisión en relación al cuidado de su salud o su tratamiento?**Por ejemplo, se decidió sobre si usted seguirá o no un tratamiento, eligió un tratamiento a seguir, o qué estudio diagnóstico o de rastreo realizar.

SI - NO

- **¿Fue informado de las diferentes opciones disponibles?**

SI - NO SÉ - NO

- **¿Fue informado acerca de los beneficios y riesgos de cada opción?**

SI - NO SÉ - NO

- **¿Fue informado acerca de cuán probables son esos beneficios y riesgos?**

SI - NO SÉ - NO

- **¿Fue consultado acerca de qué considera importante?**

SI - NO SÉ - NO

**A continuación nos gustaría que complete las siguientes preguntas:**

A- Edad: _____ años

B- Sexo (marque con una cruz lo que corresponda)

Varón ____

Mujer ____

C- Nivel educativo más alto alcanzado (Marque con una cruz lo que corresponda)

Primario completo____

incompleto____

Secundario completo

incompleto ____

Terciario completo_____

incompleto_____

Universitario completo____

incompleto _____

**Información para el investigador**

Fecha:

Nro de cuestionario:

**A continuación nos gustaría que complete las siguientes preguntas:**

A- Edad____años

B- Sexo (marque con una cruz lo que corresponda)

Varón ____

Mujer ____

C- Servicio en el que se desempeña: ______

**Información para el investigador:**

Fecha:

Nro de cuestionario:

ID:
